# Role of scrape cytology in the intraoperative diagnosis of tumor

**DOI:** 10.4103/0970-9371.71871

**Published:** 2010-07

**Authors:** Sachin S Kolte, Rahul N Satarkar

**Affiliations:** Department of Pathology, Mamata Medical College, Khammam, Andhra Pradesh, India

**Keywords:** Cytology, diagnosis, intraoperative, rapid, scrape

## Abstract

**Background:**

Rapid diagnosis of surgically removed specimens has created many controversies and a single completely reliable method has not yet been developed. Histopathology of a paraffin section remains the ultimate gold standard in tissue diagnosis. Frozen section is routinely used by the surgical pathology laboratories for intraoperative diagnosis. The use of either frozen section or cytological examination alone has an acceptable rate (93–97%) of correct diagnosis, with regard to interpretation of benign versus malignant.

**Aim:**

To evaluate the utility of scrape cytology for the rapid diagnosis of surgically removed tumors and its utilisation for learning cytopathology.

**Materials and Methods:**

75 surgically removed specimens from various organs and systems were studied. Scrapings were taken from each specimen before formalin fixation and stained by modified rapid Papanicolaou staining.

**Results:**

Of the 75 cases studied, 73 could be correctly differentiated into benign and malignant tumors, with an accuracy rate of 97.3%.

**Conclusions:**

Intraoperative scrape cytology is useful for intraoperative diagnosis of tumor, where facilities for frozen section are not available. The skill and expertise developed by routinely practicing intraoperative cytology can be applied to the interpretation of fine needle aspirate smears. Thus, apart from its diagnostic role, intraoperative cytology can become a very useful learning tool in the field of cytopathology.

## Introduction

Rapid diagnosis of surgically removed specimens has created many controversies and a single completely reliable method has not yet been developed. Histopathology of a paraffin section remains the ultimate gold standard in tissue diagnosis. Frozen section is routinely used by the surgical pathology laboratories for intraoperative diagnosis. Many studies have been done in the past to evaluate the role of cytology in intraoperative diagnosis of tumor. These studies have concluded that cytology has the advantage of being much less time consuming, easy to adopt, reliable and does not require special instruments.[1,2] Hence, scrape cytology can be employed routinely in the intraoperative diagnosis in conjunction with frozen section.[1–4] It can be used to diagnose small tissue that can be preserved for permanent paraffin block method.[5] The use of either frozen section or cytological examination alone has an acceptable rate (93–97%) of correct diagnosis, with regard to interpretation of benign versus malignant.[4–7] Commonly used methods for obtaining and preparing cells for cytological evaluation are touch preparation, fine needle aspiration cytology (FNAC) and scrape smear preparation. Scrape preparations yield cellular smears.[8,9] In addition, cytological examination of surgical specimens has proved to be a valuable learning tool and has educational value. The skill and expertise developed by routinely practicing intraoperative cytological technique can be applied to the interpretation of FNAC. This significant educational value coupled with its intrinsic simplicity and rapidity and cost effectiveness will likely necessitate the widespread implementation of this diagnostic technique in the near future.[10–12]

The present study was carried out to evaluate the utility of scrape cytology for the rapid diagnosis of surgically removed tumors.

## Materials and Methods

Gross examination of the specimen of tumor removed from the patient was done by inspection and palpation. The specimen was then cut with a sharp knife into two halves. The cut surface was wiped off the excess blood, if present, with the help of a filter paper. Again, reinspection and repalpation of the tumor was done. The most appropriate area thought to be representative of lesion was chosen. The area was scraped with a sharp scalpel or the end of a glass slide, depending upon the type of tissue. A semifluid drop thus obtained was spread over a glass slide in the same manner as FNAC. On an average, four slides per case were taken from different representative areas. The slides were labelled and immediately put into 95% ethyl alcohol and stained with a modified rapid Papanicolaou stain.

The slides were examined immediately and reported as benign or malignant. Total time taken for smear preparation, staining and reporting was about 10 minutes.

The specimens were then fixed in 10% formal saline. Sections were taken from the same area from where scrapings were taken. Paraffin blocks of the sections were processed in the routine way and 5 µm thick sections were stained with hematoxylin and eosin (H and E).

The diagnosis obtained by intraoperative scrape cytology was compared with final histopathological diagnosis in terms of diagnostic sensitivity, to differentiate between benign and malignant lesions.

## Results

**Figure 1:**
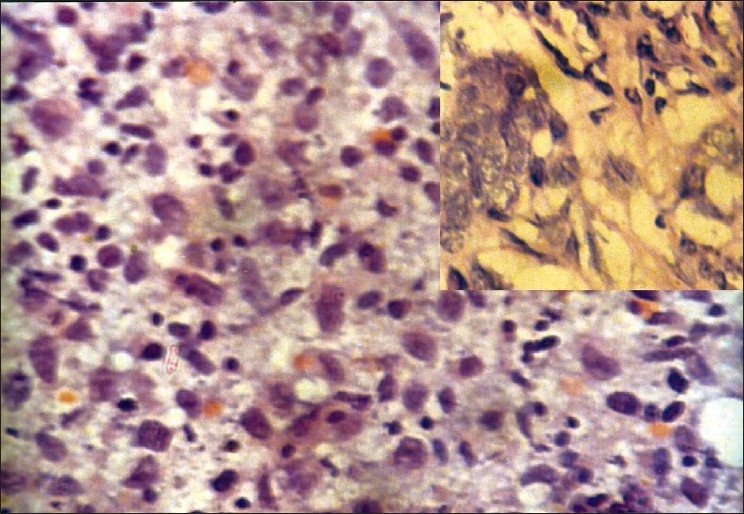
Scrape smear from infiltrating duct carcinoma of breast shows round to polygonal pleomorphic cells with hyperchromatic nuclei (Pap, ×400); inset shows histopathology of the same (H and E, ×400)

**Figure 2:**
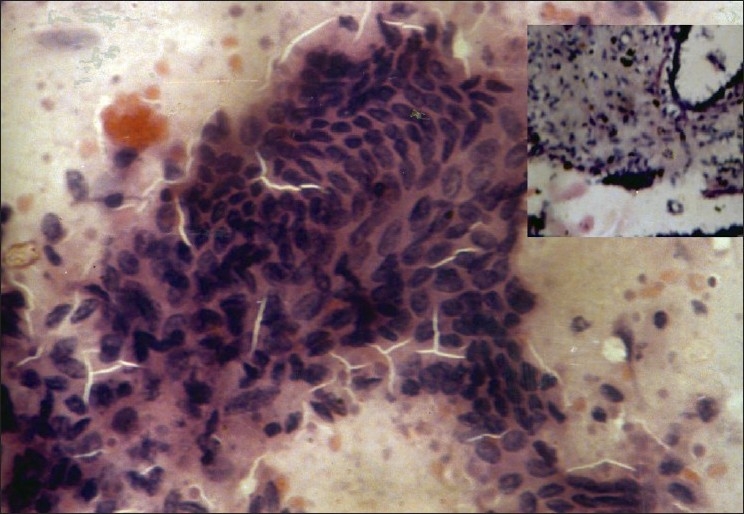
Scrape smear from fibroadenoma of breast shows tight clusters of benign epithelial cells (Pap, ×400); inset shows histopathology of the same (H and E, ×400)

**Figure 3:**
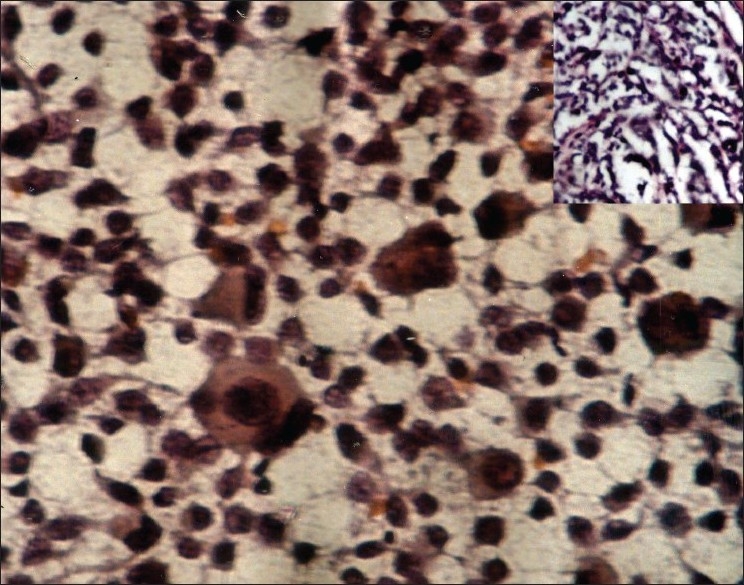
Scrape smears from sebaceous carcinoma show round cells with hyperchromatic nuclei and foamy cytoplasm, also seen are tumor giant cells (Pap, ×400); inset shows histopathology of the same (H and E, ×100)

**Figure 4:**
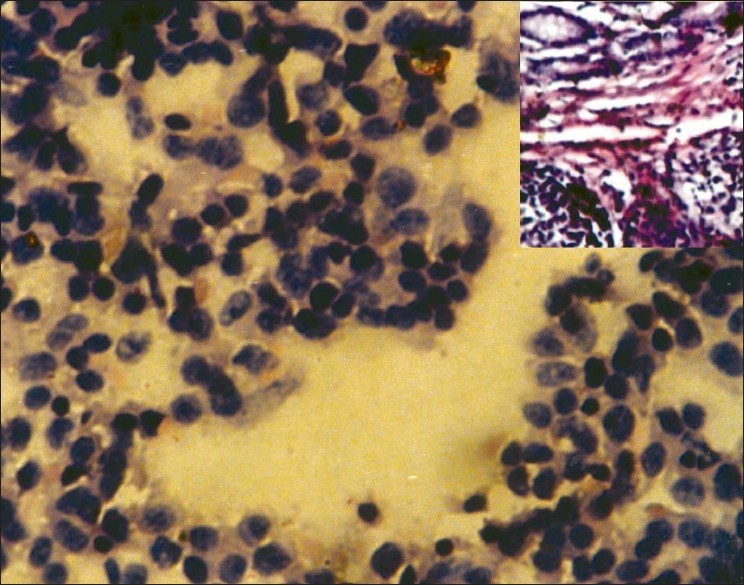
Scrape smears from lymphoma of intestine show monotonous round cells with hyperchromatic nuclei (Pap, ×400); inset shows histopathology of the same (H and E, ×100)

**Figure 5:**
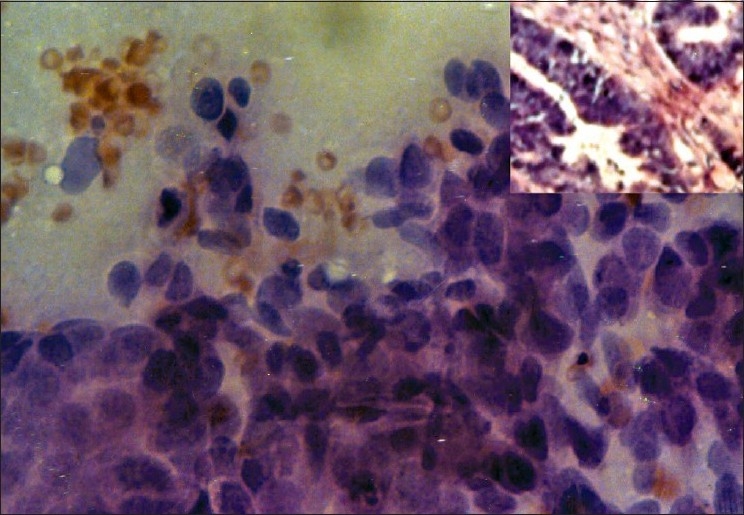
Scrape smears from adenocarcinoma of intestine shows round cells with hyperchromatic nuclei forming glandular structures (Pap, ×400); inset shows histopathology of the same (H and E, ×400)

The study included 75 surgically removed specimens [Table 1] from various sites of body such as breast, skin, soft tissues, genitourinary tract, thyroid, gastrointestinal tract, testis, and bone [Figures 1–5].

**Table 1:** Organ wise distribution of cases correctly diagnosed as benign or malignant

Organs/systems	No. of cases	Correctly diagnosed	Percentage
Breast	40	39	97.5
Skin/soft tissues	10	09	90.0
Genitourinary system	08	08	100
Thyroid	04	04	100
Gastrointestinal tract	08	08	100
Testis	03	03	100
Bone	02	02	100
Total	75	73	97.3

The distribution of cases and their diagnoses are given in Table 2. In two cases, diagnosis was not possible. One case was a breast specimen, which showed acellular smears, and cytological diagnosis was inconclusive owing to less cellularity. This turned out to be sclerosing adenosis on histopathology. Another case showed cellular smears and the opinion given was a benign lesion, but turned out to be a low-grade squamous cell carcinoma on histopathology.

**Table 2:** Distribution of cases diagnosed on cytology and histology

Organs/systems	Cytological diagnosis	Histopathological diagnosis
Breast (40)	Fibroadenoma (07)	Fibroadenoma (07)
	Inconclusive smear (01)	Sclerosing adenosis (01)
	Infiltrating ductal carcinoma (32)	Infiltrating ductal carcinoma (30)
		Infiltrating lobular carcinoma (01)
		Mucinous carcinoma (01)
Skin and soft tissue (10)	Benign spindle cell lesion (03)	Dermatofibroma (03)
	Malignant spindle cell lesion (02)	Malignant fibrous histiocytoma (01)
		Fibrosarcoma (01)
	Squamous cell carcinoma (01)	Squamous cell carcinoma (01)
	Benign squamous cell lesion (01)	Squamous cell carcinoma (01)
	Positive for malignant cells (02)	Secondaries from adenocarcinoma (01)
		Sebaceous carcinoma (01)
	Tuberculous lymphadenitis (01)	Tuberculous lymphadenitis (01)
Genitourinary system (08)	Renal cell carcinoma (02)	Renal cell carcinoma (02)
	Carcinoma bladder (01)	Carcinoma bladder (01)
	Benign hyperplasia of prostate (02)	Benign hyperplasia of prostate (02)
	Transitional cell carcinoma (kidney) (01)	Transitional cell carcinoma (kidney) (01)
	Wilm’s tumor (01)	Wilm’s tumor (01)
	Benign spindle cell lesion (01)	Leiomyoma of cervix (01)
Gastrointestinal system (08)	Tuberculosis of intestine (01)	Tuberculosis of intestine (01)
	Adenocarcinoma (04)	Adenocarcinoma (04)
	Round cell lesion (01)	Carcinoid (01)
	Lymphoma (01)	Lymphoma (01)
	Squamous cell carcinoma of oesophagus (01)	Squamous cell carcinoma of oesophagus (01)
Thyroid (04)	Colloid goitre (04)	Colloid goitre (04)
Bone (02)	Osteogenic sarcoma (02)	Osteogenic sarcoma (02)
Testes (03)	Seminoma (02)	Seminoma (02)
	Positive for malignant cells (01)	Immature teratoma (01)

Figures in parenthesis indicate the number of cases.

## Discussion

History of scrape cytology can be traced back to 1927 when Leonard S. Dudgeon and Vincent Patrick at the University of London raised the horizons of the rapid cytological diagnosis of freshly cut specimens with reliable accuracy rates. Following this, several studies done in the past have discussed the use of imprint and touch preparation, especially as a tool for intraoperative diagnosis.[1,3–5,7]

After these initial trials, the use of cytology smears during intraoperative consultation has often been neglected in favor of traditional examination of frozen sections. This appears to be due to the surgical pathologist’s relatively higher level of confidence in frozen sections, though many studies have demonstrated that the diagnostic efficacy of intraoperative cytology is comparable to that of frozen section.[6] So, this study was undertaken to know the utility of scrape cytology in the intraoperative diagnosis of tumor. We obtained very good results while using scrape cytology.

Shidham *et al*.[1] and Khunamornpong *et al*.[13] observed that scraping of tumor is the method preferred because large number of cells can be obtained and cells can be spread well on the slides. According to Esteban *et al*.,[3] touch preparation yields less cellular smears than scrape smears. We also found that smears prepared after scraping of tumor yielded uniformly cellular smears.

Sato *et al*.[14] described a modified rapid Papanicolaou stain for imprint smears. They claimed that the method was as fast as the ultrafast Papanicolaou staining, without any compromise in terms of quality of staining. We advocated the use of wet fixation and modified Papanicolaou staining, as suggested by Sato *et al*.[14] We also obtained excellent staining results so as to arrive at a diagnosis. This staining completed within 90 seconds and the whole procedure took around 10 minutes, enabling rapid diagnosis with good results as well.

Gross examination is very useful before making any impression by cytology. We studied 40 cases of breast lesions, of which 39 could be diagnosed correctly. One case showed acellular smear because of dense sclerosis. The histopathology diagnosis being sclerosing adenosis. Suen *et al*.[15] studied 473 cases of breast lesions with scrape cytology and obtained an accuracy rate of 95.7%. They noted that it was not possible to differentiate between *in situ* and infiltrative carcinoma of breast with scrape cytology.

We found that it is not difficult to diagnose malignancy of breast on cytology, but highly cellular smears of benign lesion should be carefully screened as scrape smears would yield more cellular smears. The most significant factor affecting the diagnostic accuracy of intraoperative cytology may be the number of cases in the low-grade or well-differentiated category in a particular study.[6] We did not encounter any case of low-grade infiltrating ductal carcinoma, ductal carcinoma *in situ* or atypical ductal/lobular hyperplasia. This factor may also have contributed to the high accuracy rate of our study.

Out of 10 lesions of skin and soft tissues, we diagnosed nine cases. One case of axillary mass showed less cellular smears. Smears showed few mature squamous epithelial cells. Hence, the cytological diagnosis given was benign lesion. This lesion turned out to be low-grade squamous cell carcinoma on histopathology. Desmoplastic reaction present in this tumor resulted in a lesser yield on scrape cytology, thus leading to a false negative diagnosis. Suen *et al*.[15] studied 64 cases of skin and soft tissues and diagnosed 55 cases. According to them, it is difficult to diagnose tumors with dense fibrous stroma as the number of neoplastic cells transferred to the slides is insufficient to enable to make a correct diagnosis. Suen *et al*.[15] and Kontozoglou *et al*.[12] achieved accuracy rates of 85.9 and 100%, respectively. We obtained an accuracy rate of 90%.

Our study included three cases each from small and large bowel, and one each from esophagus and stomach. All the cases were diagnosed correctly with intraoperative cytology. Three cases of testes and two cases of bone tumor were diagnosed correctly on cytology. The rates of accuracy achieved were comparable with that observed by many other authors [Table 3].

**Table 3:** Accuracy rates achieved by other authors and present study

Author	No. of cases	Accuracy (%)
Dudgeon, *et al*.[16]	200	95.5
Pickren, *et al*.[17]	1819	97.4
Mavec[18]	100	93.0
Tribe[7]	510	96.9
Suen, *et al*.[15]	108	96.3
Shidham, *et al*.[1]	249	98.4
Esteben, *et al*.[3]	140	87.5
Kontozoglou, *et al*.[12]	215	99.1
Present study	075	97.3

Intraoperative cytology has high accuracy rates, excellent preservation of cellular details, and the possibility of identifying focal, macroscopically undetectable neoplastic lesion in large tissue fragments. The method is simple and inexpensive, not requiring special technique or instrument. At the centers where the facilities of frozen section are not available, intraoperative scrape cytology is a useful tool for intraoperative diagnosis of tumor.

The disadvantages of intraoperative cytology are very few and high accuracy rates can be achieved with experience. It is though not possible to distinguish *in situ* from infiltrating carcinoma and to evaluate the depth of invasion and/or margins of resection.

In recent times, the role of FNAC has increased as the same is done from various sites under image guidance. In such a scenario, the material obtained from intraoperative scrape cytology can be interpreted as FNAC smears for better training in cytopathology.[19]

Thus, apart from its diagnostic role, intraoperative cytology can become a very useful learning tool. It can promote interpretation of cytology smears and its histological correlation, as the material obtained can be interpreted as FNAC smears.

This study has included surgically removed specimens, and hence, in some cases, the diagnosis was arrived preoperatively either by FNAC or incisional biopsy. Also the study is limited by small sample size.
